# Stress Responses of Shade-Treated Tea Leaves to High Light Exposure after Removal of Shading

**DOI:** 10.3390/plants9030302

**Published:** 2020-03-01

**Authors:** Satoshi Sano, Tetsuyuki Takemoto, Akira Ogihara, Kengo Suzuki, Takehiro Masumura, Shigeru Satoh, Kazufumi Takano, Yutaka Mimura, Shigeto Morita

**Affiliations:** 1 Graduate School of Life and Environmental Sciences, Kyoto Prefectural University, Kyoto 606-8522, Japan; 2 Agriculture and Forestry Technology Department, Kyoto Prefectural Agriculture, Forestry and Fisheries Technology Center, Kameoka, Kyoto 621-0806, Japan; 3 Biotechnology Research Department, Kyoto Prefectural Agriculture, Forestry and Fisheries Technology Center, Seika, Kyoto 619-0244, Japan; 4 Faculty of Agriculture, Ryukoku University, Seta, Otsu 520-2194, Japan

**Keywords:** *Camellia sinensis*, covering culture, high light, oxidative stress, shading treatment, tea

## Abstract

High-quality green tea is produced from buds and young leaves grown by the covering-culture method, which employs shading treatment for tea plants (*Camellia sinensis* L.). Shading treatment improves the quality of tea, but shaded tea plants undergo sudden exposures to high light (HL) at the end of the treatment by shade removal. In this study, the stress response of shaded tea plants to HL illumination was examined in field condition. Chl *a*/*b* ratio was lower in shaded plants than nonshaded control, but it increased due to exposure to HL after 14 days. Rapid decline in *F*v/*F*m values and increases in carbonylated protein level were induced by HL illumination in the shaded leaves on the first day, and they recovered thereafter between a period of one and two weeks. These results revealed that shaded tea plants temporarily suffered from oxidative damages caused by HL exposure, but they could also recover from these damages in 2 weeks. The activities of antioxidant enzymes, total ascorbate level, and ascorbate/dehydroascorbate ratio were decreased and increased in response to low light and HL conditions, respectively, suggesting that the upregulation of antioxidant defense systems plays a role in the protection of the shaded tea plants from HL stress.

## 1. Introduction

The tea plant (*Camellia sinensis* (L.) O. Kuntze) is an important commercial beverage crop cultivated worldwide. Its apical buds and young leaves are harvested and used for producing tea. The quality of the produced tea is affected by various metabolites that contribute to its taste and flavor. Those compounds include amino acids, particularly theanine, and catechins that provide tea with good and astringent taste, respectively [1,2]. Catechins possess antioxidant and anticancer activities [3], thereby providing the tea with beneficial effects on human health; theanine produces a relaxing and an arousal effect by increasing alpha-band activity in the human brain [4].

Tea is mainly consumed as green tea in Japan, and high-quality green tea such as Matcha and Gyokuro is produced from the buds and leaves of the plant grown by the covering-culture method, which has been established in the Uji area located to the south of Kyoto [5]. In this traditional cultivation method, tea trees are covered with shading nets when new shoots emerge, and they are grown under the shade before being harvested after between 14 and 30 days. During this period, the contents of chlorophylls (Chls) and free amino acids including theanine are increased, and that of catechins is decreased, in the tea shoot [6,7,8,9,10,11]. Tea trees usually produce new shoots thrice in a year from spring to fall in Japanese tea plantations. The harvest obtained from shoots produced in April and May is called the first crop and that obtained in summer is called the second crop. Shading was initially performed only in the first cropping season, but this process is now being applied to the second cropping season as well to meet the growing demand for Matcha (fine powdered green tea) in the last two decades [12]. However, using shading treatment in both the first and second cropping season for many years negatively affects tea trees. A previous study reported that consecutive shading treatments in the first and second cropping season resulted in the suppression of growth of new shoots in the subsequent season, eventually decreasing tea shoot yield [12].

It is likely that the suppression of shoot growth caused as a result of long-term shading treatment can be attributed to a reduction in photosynthetic products under low light (LL) condition, but shading treatment may also affect tea plants in different manners. Since the tea trees are suddenly exposed to high light (HL) by shade removal at the end of shading treatment, they are repetitively exposed to stresses due to HL exposure by consecutive shading treatments in a year. HL illumination is deleterious to plants since excess light energy enhances the production of reactive oxygen species (ROS), which in turn causes photooxidative damage to plant cells [13,14]. Under HL conditions, superoxide anion radical (O_2_^−^) is produced in chloroplasts through the reduction in molecular oxygen (O_2_) in the photosystem (PS) I, and this O_2_^−^ disproportionates to hydrogen peroxide (H_2_O_2_) and O_2_. ROS including O_2_^−^ and H_2_O_2_ lead to cellular damage by the oxidation of biomolecules such as nucleic acids, proteins, and lipids. For protection against ROS, plants are equipped with antioxidant defense systems which consist of ROS scavenging enzymes and low molecular weight antioxidants [13,14]. The detoxification of O_2_^−^ is catalyzed by superoxide dismutase (SOD), and H_2_O_2_ is scavenged mainly by ascorbate peroxidase (APX) using ascorbate (AsA) in plants. In the APX reactions, AsA is oxidized to monodehydroascorbate (MDA) radical, and MDA disproportionates to AsA and dehydroascorbate (DHA). DHA is unstable at physiological pH and is decomposed irreversibly by spontaneous and enzymatic reactions [15]. AsA is an essential antioxidant which plays a central role in protection against oxidative stress in plant cells. Therefore, MDA and DHA, which are generated by the reaction of APX as well as the nonenzymatic quenching of O_2_^−^, must be reduced to maintain the pool of AsA in cells. MDA reductase (MDAR), DHA reductase (DHAR) and glutathione reductase (GR) are involved in the regeneration of AsA using reducing power from the electron transport chain in chloroplasts [13,14,16].

Moreover, abiotic stresses such as chilling, heat, and drought enhance ROS production, leading to oxidative damage to plants [14]. In tea plants, ROS accumulation during winter dormancy and increased lipid peroxidation under cold stress have been documented [17,18]. ROS accumulation, oxidative damages, and upregulation of antioxidative defense systems have been observed under cold, salt, and drought stresses [19]. The responses of genes and proteins involved in AsA synthesis and metabolism under temperature stress have also been examined in tea plants [20,21]. In addition, the response of antioxidative enzymes to shading has been studied [22]. However, the effects of stresses associated with shade removal in shaded tea trees have not been studied to date. In this study, we examined the physiological changes in shaded tea plants after shade removal in the field condition and found that tea plants temporarily suffer from oxidative damage caused by sudden HL illumination after shading treatment.

## 2. Results

### 2.1. Long-Term Shading Treatment of Tea in Field Condition

In this study, we performed shading treatment of tea plants in the field by covering them directly with shading nets for 30 days in the first cropping season and 20 days in the second cropping season, respectively. The experiment started in the first cropping season of 2011; shading treatment continued in both the first and the second cropping seasons for 5 years [12]. The number, length, and weight of shoots decreased in the fall season of 2012, which eventually resulted in decreased yield, and the number of shoots and yield also decreased in the first cropping season in 2013 [12], indicating the negative impact of consecutive shade treatments after the second cropping season in the second year.

### 2.2. Changes in Chl Content and Chl a/b Ratio

In order to examine the effects of HL illumination after shading treatment, we monitored the physiological changes of shaded tea plants for 14 days after shade removal in the first and the second cropping season of 2012. The daily high, low and mean temperatures, and hours of sunlight during the study period are shown in Figures S1 and S2. Photosynthetic photon flux densities (PPFD) on the canopy of the tea trees was 1300–2000 µmol m^−2^ s^−1^ on sunny days, and 300–1000 µmol m^−2^ s^−1^ on cloudy and rainy days.

**Figure 1:**
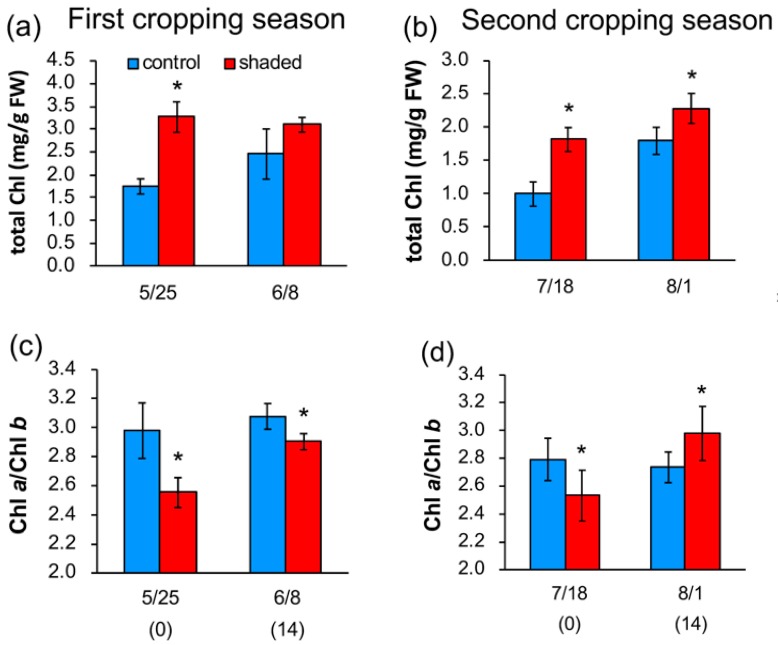
Changes in total Chl content (**a**,**b**) and Chl *a*/*b* ratio (**c**,**d**) in shaded tea leaves after shade removal. Data from the first (**a**,**c**) and second (**b**,**d**) cropping seasons are shown as means ± standard deviation (*n* = 5). The *x*-axis indicates the date of sampling, and numbers in parentheses show the days after shade removal in shaded plants. Asterisks indicate significant differences between the control and shaded plants (*p* < 0.05, *t*-test).

It has been reported that shading treatment of tea plants increases Chl levels [6,7,8,10,11]. In our experiments, total Chl content was increased by shading treatment, as shown by the data at day 0 in Figure 1a,b. The Chl content in shaded leaves (0 days after shade removal) was higher than that in the control leaves by 87% and 82% in the first and second cropping seasons, respectively. Total Chl level increased in the control leaves during 14 days and reached a similar level to that in shaded leaves at 14 days in the first crop season. Chl content increased both in the control and shaded plants in the second cropping season, but their difference was smaller at 14 days than at 0 days.

Higher plants acclimate to the LL condition by increasing the level of light-harvesting chlorophyll *a*/*b* proteins, resulting in a decrease in Chl *a*/*b* ratio [23]. Since the Chl *a*/*b* ratio is an index of LL acclimation, we measured Chl *a*/*b* ratio in the leaves of shaded tea plants. Chl *a*/*b* ratio was significantly lower in shaded leaves than in the control leaves both in the first and second cropping seasons (Figure 1c,d), which was consistent with trends in a previous study of shading treatment of tea plants [11]. This result suggests that shaded tea plants acclimated to LL conditions by increasing the size of light-harvesting antenna. The ratio in the shaded plants increased after shade removal in response to HL illumination in both the first and second cropping season (Figure 1c,d). Although the difference between the control and shaded leaves was significant, it was smaller at 14 days than at 0 days in the first cropping season. Chl *a*/*b* ratio was even higher in the shaded leaves than in the control leaves after 14 days in the second cropping season. These results indicate that shaded tea plants can acclimate to HL conditions in 14 days.

### 2.3. Changes in Chl Fluorescence

**Figure 2:**
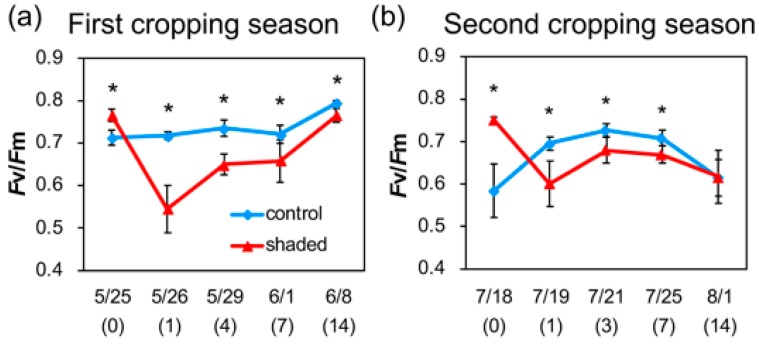
Changes in Chl fluorescence in shaded tea leaves after shade removal. Data from the first cropping season (**a**) and second cropping season (**b**) are shown as means ± standard deviation (*n* = 6–8). The x axis indicates the date of sampling, and numbers in parentheses show the days after shade removal in the shaded plants. Asterisks indicate significant differences between the control and shaded plants (*p* < 0.05, *t*-test).

We measured the Chl fluorescence of PS II in the leaves of shaded plants and monitored changes in *F*v/*F*m, which is an index of photochemical activity of PS II [24], in response to HL illumination (Figure 2). At the end of the shading treatment (0 days after shade removal), shaded leaves showed higher *F*v/*F*m values than the control leaves in both first and the second cropping season. This difference may be attributed to the acclimation of shaded leaves to LL. Alternatively, this can be attributed to the suppression of PS II activity in nonshaded leaves, since PS II activity is downregulated under HL conditions due to the dissipation of excess energy [25,26]. The net photosynthesis rate of tea leaves was reported to be the highest at 1200 μmol m^−2^ s^−1^ PPFD, and it reduced with increased PPFD under the field conditions [27]. This result suggests that photosynthesis is suppressed under HL conditions in field-grown tea plants, which is consistent with our current observation.

After shade removal, *F*v/*F*m values were decreased in shaded leaves on the first day to 71% and 80% in the first and the second cropping season, respectively. However, *F*v/*F*m values in shaded leaves were increased thereafter and recovered to a similar level to that in the control leaves after 14 days. The rapid decline in *F*v/*F*m values indicates the inhibition of PS II under HL illumination [26]. It was suggested that excess light energy was absorbed by the elevated level of light-harvesting antenna in LL-acclimated leaves when the shaded plants were suddenly exposed to HL illumination following shade removal, which may lead to an overreduction in photosynthetic electron transport chain and the enhancement of ROS production. Therefore, LL-acclimated leaves of shaded tea plants with an increased level of antenna can be damaged by the illumination of sunlight that is not harmful to nonshaded leaves. PSII activity is reduced under HL stress by an imbalance of degradation and repair of D1 protein [28]. Since ROS inhibit the repair of PSII by suppressing the synthesis of D1 protein [29,30], the photodamage of PSII caused by elevated ROS production by HL exposure is likely reflected in the decreased *F*v/*F*m observed in our study.

Our results also indicate that reduced *F*v/*F*m in shaded leaves can recover in two weeks, which suggests that D1 protein is repaired during this period. Although the turnover of D1 protein is known to be rapid [28], recovery of *F*v/*F*m was slow in the tea plants and it took two weeks for the shaded plants to restore PSII activity. It was assumed that the acclimation process for downregulation of antenna proteins in response to HL takes time and that the shaded plants were subjected to HL stress until the antenna size decreased to a similar level to that of control plants. The data for Chl *a*/*b* ratios indicate that HL acclimation of the shaded leaves occurred within 14 days (Figure 1c,d), which coincides with the two-week recovery period for *F*v/*F*m.

### 2.4. Changes in Carbonylated Protein Content

**Figure 3:**
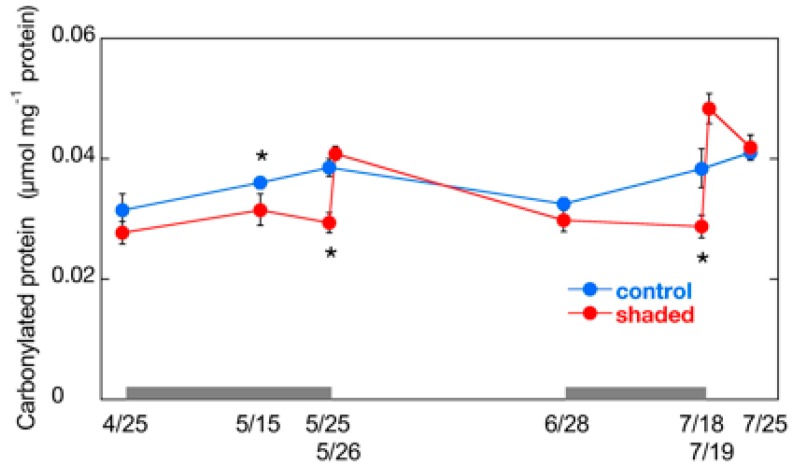
Changes in carbonylated protein content in shaded tea leaves after shade removal. Gray bars indicate the period of shading treatments. Data are shown as means ± standard deviation (*n* = 3). Statistical analysis was performed by pairwise comparison of control and shaded plants by *t*-test. The data 1 day after shade removal in the shaded plants were compared with those at day 0 in the control. Asterisks indicate significant differences (*p* < 0.05).

In order to evaluate oxidative injuries in shaded leaves, we measured the content of carbonylated proteins, which is an index of oxidative stress [31]. Various proteins involved in important cellular processes, such as subunits of PSII [32], thiol enzymes in Calvin cycle [33], and some mitochondrial proteins [34], are carbonylated. Carbonylated protein content was significantly lower in shaded leaves (approximately 75% in the both cropping seasons) than in the control during shading treatments (Figure 3). The content was increased sharply after shade removal in shaded leaves. The level of carbonylated proteins in shaded leaves was similar to that in the control after the first day in the first cropping season, and it was higher (1.3 times of the control) in the second cropping season. This result suggests that sudden exposure to HL imposed oxidative stress on the shaded leaves in summer season. The carbonylated protein content in shaded leaves of the second cropping season was decreased thereafter, and was similar to the level in the control leaves after 7 days, which reflects the degradation of carbonylated proteins by proteases [35].

### 2.5. Changes in Antioxidative Enzyme Activities

Our results reveal that shaded tea plants can recover from HL-induced damage in two weeks, which suggests that antioxidant defense systems might efficiently function for recovery from the damages. Therefore, we next examined the changes in ROS scavenging systems in the shaded leaves.

**Figure 4:**
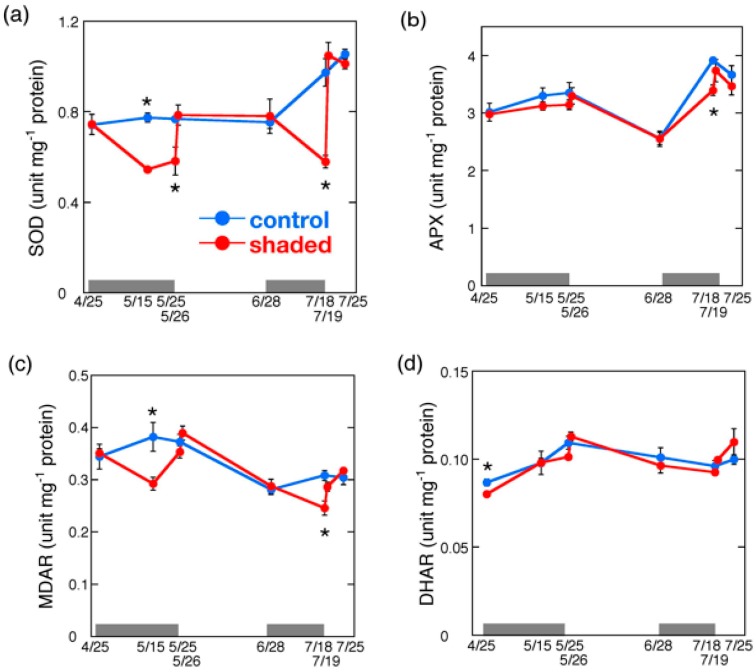

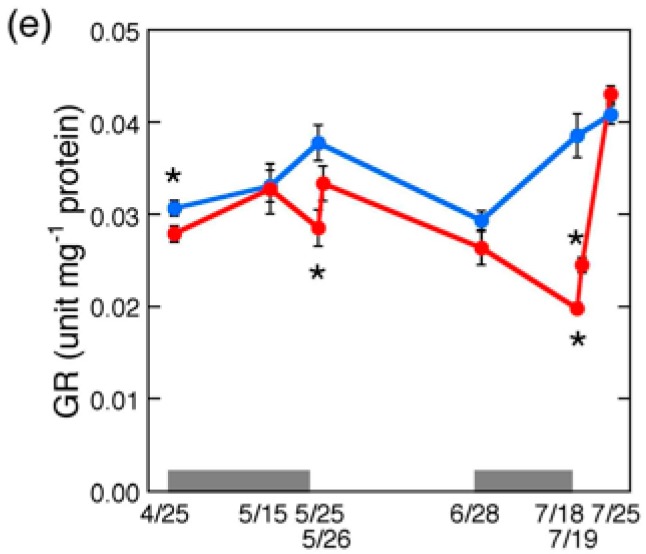
Changes in ROS scavenging enzyme activity in shaded tea leaves after shade removal. Specific activities of superoxide dismutase (SOD) (**a**), ascorbate peroxidase (APX) (**b**), MDA reductase (MDAR) (**c**), DHA reductase (DHAR) (**d**), and glutathione reductase (GR) (**e**) are shown as means ± standard deviation (*n* = 3). Gray bars indicate the period of shading treatments. Statistical analysis was performed as described in Figure 3. Asterisks indicate significant differences (*p* < 0.05).

SOD activity was significantly decreased in the shaded leaves during shading treatment (Figure 4a). It was approximately 60% of the control level at the end of shading treatment of both cropping seasons. Shade removal increased the SOD activity to a similar level to that of the control leaves after the first day. MDAR activity showed a similar trend to SOD, in which the activity was reduced during shading treatment and increased in response to shade removal (Figure 4c). GR activity was also reduced by shading treatment, and increased by HL exposure, although it did not reach the same level as in the control after the first day (Figure 4e). In contrast, APX and DHAR activities showed little change during shading treatment and subsequent HL illumination (Figure 4b,d). The changes in the activities of SOD and MDAR were consistent with the light intensities of cultivation, which suggests that the activities of these enzymes are regulated in response to ROS production. It is suggested that the increase in SOD and MDAR activities contribute to the elevation of ROS scavenging capacity, protection, and recovery of shaded leaves from oxidative injuries under HL condition.

### 2.6. Changes in AsA Content and Its Redox Level

**Figure 5:**
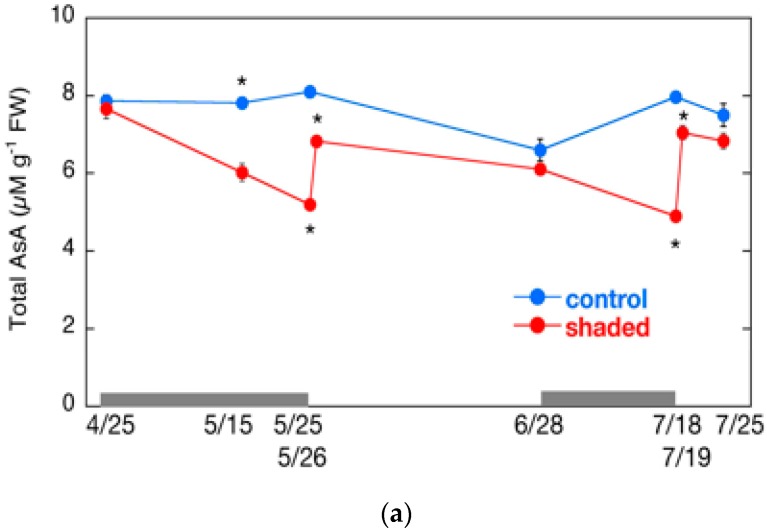

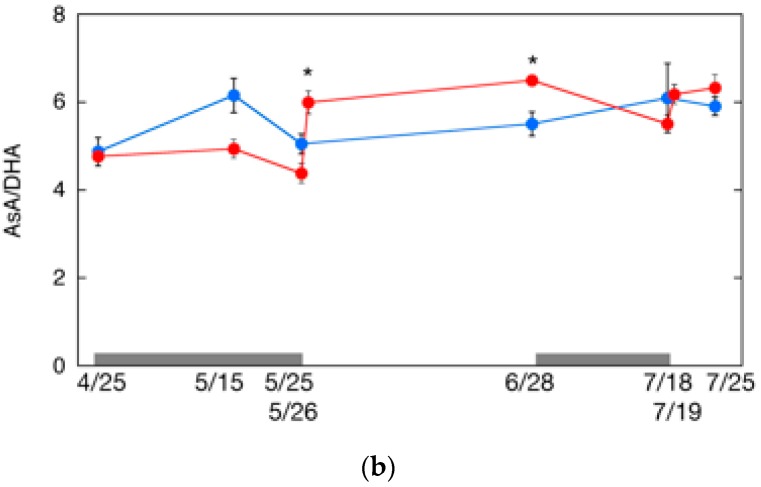
Changes in AsA content in shaded tea leaves after shade removal. (**a**) Total AsA content and (**b**) the ratio of AsA to DHA are shown. Gray bars indicate the period of shading treatments. Data are shown as means ± standard deviation (*n* = 3). Statistical analysis was performed as described in Figure 3. Asterisks indicate significant differences (*p* < 0.05).

We also measured the AsA content and the ratio of AsA to DHA. The AsA content fluctuated during shading treatment and subsequent HL exposure (Figure 5a). The fluctuation was similar to that of the carbonylated proteins mentioned above. The total AsA content declined significantly to approximately 60% of the control level during shading treatment in both cropping seasons. The content in the shaded leaves increased sharply after the shade removal, but the AsA content after the first day did not reach that of the control leaves in both cropping seasons. Similarly, the AsA/DHA ratio in the shaded tea leaves decreased during the shading treatment, and increased after the shade removal (Figure 5b).

Previous studies demonstrate that shading decreases AsA levels in rainforest trees [36] and HL increases them in three evergreen plant species [37]. The expression of genes involved in AsA biosynthesis is induced by continuous light in *Arabidopsis* [38,39]. It is likely that the decrease in AsA content and AsA/DHA ratio by the shading treatment is due to decreased MDAR and GR activities (Figure 4c,e), which are responsible for AsA regeneration. The regeneration of AsA may also be lowered by the decrease in the supply of the reducing power from the electron transport chain under LL conditions. After shade removal, the total AsA content did not reach that of the control level at day 1 in the shaded plants (Figure 5a), suggesting that the induction of AsA biosynthesis enzymes was not high enough, although the plants were stressed by HL exposure. In contrast, HL exposure increased the AsA/DHA ratio, likely due to the induction of MDAR and GR (Figure 4c,e), and the elevation of the supply of the reducing power in chloroplasts.

## 3. Discussion

Our results suggest that shaded tea plants acclimate to LL conditions by increasing their light-harvesting efficiency during shading treatment. The sudden exposure of shaded plants to HL caused a decrease in *F*v/*F*m and an increase in carbonylated protein levels on the first day after shade removal. The decrease in *F*v/*F*m may reflect the downregulation of PSII as a defense mechanism against HL. The activity of PSII is inhibited under HL conditions, but PSI activity remains high in *Arabidopsis* [40]. Tikkanen et al. (2014) propose that the inhibition of PSII contributes to the protection of PSI from photodamage [41]. If so, the decrease in *F*v/*F*m observed in the shaded tea leaves may function as a protection against HL. However, we found elevated carbonylated protein levels, which indicate oxidative injuries caused by HL. It is therefore likely that HL exposure caused the enhanced production of ROS from excess light energy absorbed by LL-acclimated PS, although downregulation of PSII can occur in shaded leaves. In addition, antioxidant systems were downregulated, and the supply of the reducing power required for scavenging ROS was supposed to decrease under LL conditions. In this situation, it is plausible that elevated ROS were not scavenged efficiently immediately after shade removal, which resulted in oxidative damage to PSII and cellular proteins.

Our data also reveal that antioxidant defense systems were induced rapidly one day after shade removal, which enhanced ROS scavenging and recovery of proteins from oxidative injury in 1 week. It is suggested that HL acclimation decreased light absorption and ROS production by downregulating antenna proteins. These processes contributed to the repair of PSII, and PSII activity was restored after 14 days.

LL-acclimated tea plants suffered from photooxidative stress by HL exposure following shade removal. However, the oxidative injuries caused by HL were temporary, and the tea plants were able to recover in two weeks. Since the damages observed in this study did not last for a long period, it is unlikely that the photooxidative stress after shade removal affects the shoot growth in the subsequent season and in the next year. We could not elucidate the mechanism underlying the negative impact of long-term shading treatment in this study, which will be a subject of future study. Decreased photosynthesis due to long-term shading treatment likely affects primary metabolism, which may, in turn, lead to changes in the metabolome of shaded tea leaves. A detailed study of changes in metabolites might help predict the growth of tea shoots in future.

This study revealed the stress response of shaded tea leaves to HL exposure for the first time. Our results suggest that the elevation of the activities of ROS scavenging enzymes plays a role in the defense against HL exposure. In addition, various defense mechanisms other than ROS scavenging system also contribute to the acclimation to HL after shade removal. These mechanisms include heat dissipation by non-photochemical quenching [42], cyclic electron flow [43], and P700 oxidation system [44], which were not examined in this study, in addition to a number of stress responsive genes/proteins such as heat shock factors and heat shock proteins [45,46]. In addition, HL and abiotic stress responses are mediated by hormonal regulation [47,48], which may also be the case in the HL response of shaded tea plants. Comprehensive analyses of stress responses of tea plants using omics approach will clarify the molecular basis of those systems in future study.

## 4. Materials and Methods

### 4.1. Plant Material and Growth Conditions

Tea plants (cv. ‘Yabukita’, 38 years old) grown in the experimental field of Tea Industry Research Division, Agriculture and Forestry Technology Department, Kyoto Prefectural Agriculture, Forestry and Fisheries Technology Center (Uji, Kyoto, Japan, 34°53′ N, 135°49′ E, elevation: 84 m) were used in this study. We set open-culture plots, where tea trees were grown under full sunlight as control plants, and shaded plots where plants were grown under shaded condition for 30 days in the first cropping season and 20 days in the second cropping season twice a year for 5 years from 2011 and 2015. Shading treatment started when the second leaves had emerged, and was performed by directly covering the tea canopies with a black polyethylene net (shade level: 85%) for 30 days in the first cropping season and 20 days in the second cropping season, respectively. The period of the shading treatments in 2011 and 2012 are shown in Table S1. Weather condition (temperatures and sunlight) in the research field was monitored with a meteorological observation system (Ogasawara Keiki, Tokyo, Japan) during the study period.

On the day of shade removal when the shade treatment was completed, Chl fluorescence was measured in the shaded leaves under the net cover just before shade removal. The shade was removed at 12:15–12:30 p.m., and then leaves were sampled for the following assays.

### 4.2. Measurement of Chl Fluorescence

The Chl fluorescence of PS II was measured with a handheld chlorophyll fluorometer (model OS30p, Opti-Sciences, Inc., Hudson, NH, USA). The second or the third leaves from apical buds that had fully developed were covered and dark-adapted for 15 min prior to the measurement with leaf clips supplied by the manufacturer. Then, *F*_O_ was measured with a modulated actinic light and *F*m was determined after saturating light flash. The maximal quantum yield of PS II (*F*v/*F*m) was calculated from *F*v = *F*m − *F*_O_.

### 4.3. Measurement of Chl Contents

The first or the second leaves were collected from the trees, and two circular leaf discs (c.a. 30 mg) were prepared from both side of the main vein of the leaves, and soaked in 100% acetone at 4 °C in the dark for more than 3 days to aid extraction. The remaining Chls were then extracted by grinding the leaf discs in 100% acetone, and the extracts were recovered after centrifugation at 20,000× *g* for 3 min at room temperature. Both extracts were combined and diluted with water to 80% acetone. Total Chl contents and the ratio of Chl *a* to Chl *b* were measured according to Arnon [49].

### 4.4. Spectrophotometric Determination of Carbonylated Proteins

Apical buds and first two leaves from shoot apex were sampled and used for the measurement of carbonylated protein level, activities of antioxidative enzymes, and the AsA and DHA content. The samples were frozen with liquid nitrogen, crushed into pieces, and stored at −80 °C until use.

For carbonylated proteins quantification, the reaction of carbonyls with 2, 4-dinitrophenylhydrazine (DNPH) was used as described previously [50].

The frozen samples were ground to fine powder with a mortar and pestle in liquid nitrogen. The leaf powder (c.a. 0.7 g) were homogenized with 1.4 mL of 10 mM sodium phosphate buffer (pH 7.4) containing 1% Triton X-100, 2 mM EDTA and 1 mM phenylmethane sulfonyl fluoride (PMSF). The homogenates were centrifuged at 1800× *g* for 10 min, and 200 µL of supernatants were mixed with 300 µL of 10 mM DNPH in 2 M HCl. For the blank, the same volume of supernatants was mixed with 300 µL of 2 M HCl. After 1 h of dark incubation at room temperature with vigorous mixing at 10 min intervals, proteins were precipitated with 10% (*w*/*v*) trichloroacetic acid (TCA). The pellets were washed three times with 500 µL of ethanol/ethylacetate (1/1) by centrifugation at 1800× *g* for 5 min. The pellets were finally dissolved in 1 mL of 6 M guanidine hydrochloride in 20 mM potassium phosphate buffer (pH 2.3) and the absorption at 370 nm was measured.

### 4.5. Assay of Antioxidative Enzymes

The frozen samples were ground to fine powder with a mortar and pestle in liquid nitrogen, and the leaf powder (c.a. 0.7 g) were homogenized with 1.4 mL of 50 mM HEPES-KOH (pH 7.6) containing 1% (*v*/*v*) Triton X-100, 2 mM EDTA and 10% (*v*/*v*) polyvinyl pyrrolidone. For the assay of APX, 5 mM AsA were added to the buffer. The homogenate was centrifuged at 1800× *g* at 4 °C for 10 min and the supernatant was used for enzyme assays. For SOD, low molecular weight compounds were removed from the extract with a HiTrap Desalting column (GE Healthcare, Little Chalfont, UK). The activities of SOD [51], APX [52], MDAR [53], DHAR [54] and GR [55] were measured as previously described.

The concentration of protein was determined according to Bradford [56] using bovine serum albumin as the standard.

### 4.6. The Determination of AsA Content

The frozen samples were ground to fine powder with a mortar and pestle in liquid nitrogen, and the leaf powder (c.a. 0.7 g) were homogenized with 1.4 mL of 6% (*w*/*v*) TCA. The homogenate was centrifuged at 1800× *g* for 10 min at 4 °C. A volume of the supernatant and two volumes of 0.2 M potassium phosphate buffer (pH 7.4) were mixed to give a pH of about 6.0. The neutralized extract was immediately applied for the determination of AsA and DHA as described previously [57].

## Supplementary Materials

The following are available online at https://www.mdpi.com/2223-7747/9/3/302/s1, Figure S1: Daily temperatures and hours of sunlight during the study period in the first cropping season in 2012, Figure S2: Daily temperatures and hours of sunlight during the study period in the second cropping season in 2012, Table S1: The shade treatment period of tea plants between 2011 and 2012.
