# Supplementary material for: Stress Responses of Shade-Treated Tea Leaves to High Light Exposure after Removal of Shading
Source: Plants (Basel). 2020 Mar 1;9(3):302. doi: 10.3390/plants9030302 (PMC7154902; doi:10.3390/plants9030302)
Supplement: Supplementary file 1 [file plants-09-00302-s001.zip › supplementary files_plants-706262_R3/Table_S1_200210.docx]

**Table S1.** The shade treatment period of tea plants between 2011 and 2012.

| **Year** | **Cropping season** | **Period** | **Duration** |
| --- | --- | --- | --- |
| 2011 | First | April 26 – May 26 | 30 days |
|  | Second | July 8 – July 28 | 20 days |
| 2012 | First | April 25 – May 25 | 30 days |
|  | Second | June 28 – July 18 | 20 days |
