# Supplementary material for: Stress Responses of Shade-Treated Tea Leaves to High Light Exposure after Removal of Shading
Source: Plants (Basel). 2020 Mar 1;9(3):302. doi: 10.3390/plants9030302 (PMC7154902; doi:10.3390/plants9030302)
Supplement: Supplementary file 1 [file plants-09-00302-s001.zip › supplementary files_plants-706262_R3/FigS1&S2_200210.pptx]

## Slide 1
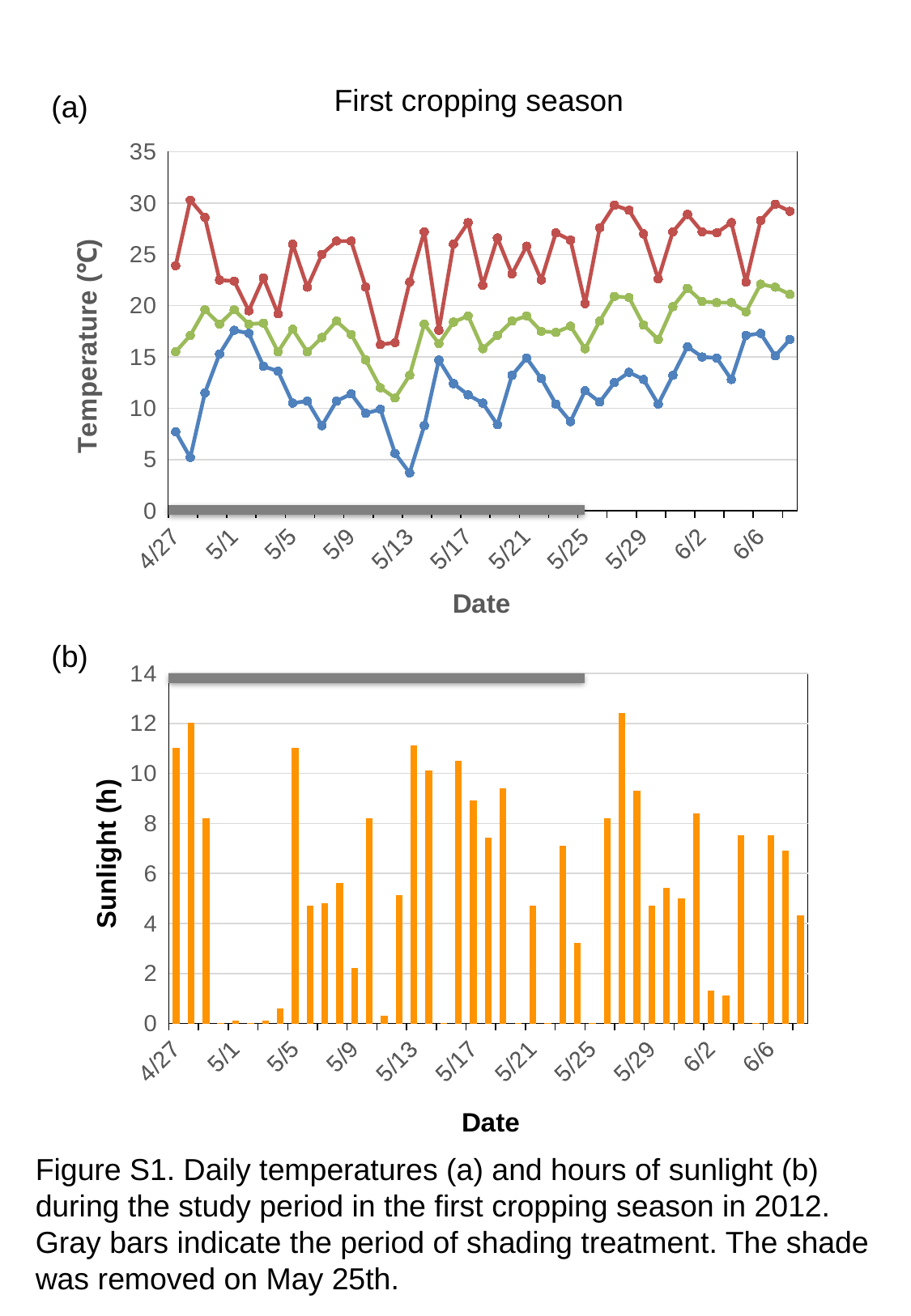

First cropping season
(a)
### Chart
| Category | Mean | Maximum | Minimum |
|---|---|---|---|
| 41026 | 15.5 | 23.9 | 7.7 |
| 41027 | 17.1 | 30.3 | 5.2 |
| 41028 | 19.6 | 28.6 | 11.5 |
| 41029 | 18.2 | 22.5 | 15.3 |
| 41030 | 19.6 | 22.4 | 17.6 |
| 41031 | 18.2 | 19.5 | 17.3 |
| 41032 | 18.3 | 22.7 | 14.1 |
| 41033 | 15.5 | 19.2 | 13.6 |
| 41034 | 17.7 | 26.0 | 10.5 |
| 41035 | 15.5 | 21.8 | 10.7 |
| 41036 | 16.9 | 25.0 | 8.3 |
| 41037 | 18.5 | 26.3 | 10.7 |
| 41038 | 17.2 | 26.3 | 11.4 |
| 41039 | 14.7 | 21.8 | 9.5 |
| 41040 | 12.0 | 16.2 | 9.9 |
| 41041 | 11.0 | 16.4 | 5.6 |
| 41042 | 13.2 | 22.3 | 3.7 |
| 41043 | 18.2 | 27.2 | 8.3 |
| 41044 | 16.3 | 17.6 | 14.7 |
| 41045 | 18.4 | 26.0 | 12.4 |
| 41046 | 19.0 | 28.1 | 11.3 |
| 41047 | 15.8 | 22.0 | 10.5 |
| 41048 | 17.1 | 26.6 | 8.4 |
| 41049 | 18.5 | 23.1 | 13.2 |
| 41050 | 19.0 | 25.8 | 14.9 |
| 41051 | 17.5 | 22.5 | 12.9 |
| 41052 | 17.4 | 27.1 | 10.4 |
| 41053 | 18.0 | 26.4 | 8.7 |
| 41054 | 15.8 | 20.2 | 11.7 |
| 41055 | 18.5 | 27.6 | 10.6 |
| 41056 | 20.9 | 29.8 | 12.5 |
| 41057 | 20.8 | 29.3 | 13.5 |
| 41058 | 18.1 | 27.0 | 12.8 |
| 41059 | 16.7 | 22.6 | 10.4 |
| 41060 | 19.9 | 27.2 | 13.2 |
| 41061 | 21.7 | 28.9 | 16.0 |
| 41062 | 20.4 | 27.2 | 15.0 |
| 41063 | 20.3 | 27.1 | 14.9 |
| 41064 | 20.3 | 28.1 | 12.8 |
| 41065 | 19.4 | 22.3 | 17.1 |
| 41066 | 22.1 | 28.3 | 17.3 |
| 41067 | 21.8 | 29.9 | 15.1 |
| 41068 | 21.1 | 29.2 | 16.7 |(b)
### Chart
| Category | Sunlight (h) |
|---|---|
| 41026 | 11.0 |
| 41027 | 12.0 |
| 41028 | 8.2 |
| 41029 | 0.0 |
| 41030 | 0.1 |
| 41031 | 0.0 |
| 41032 | 0.1 |
| 41033 | 0.6 |
| 41034 | 11.0 |
| 41035 | 4.7 |
| 41036 | 4.8 |
| 41037 | 5.6 |
| 41038 | 2.2 |
| 41039 | 8.2 |
| 41040 | 0.3 |
| 41041 | 5.1 |
| 41042 | 11.1 |
| 41043 | 10.1 |
| 41044 | 0.0 |
| 41045 | 10.5 |
| 41046 | 8.9 |
| 41047 | 7.4 |
| 41048 | 9.4 |
| 41049 | 0.0 |
| 41050 | 4.7 |
| 41051 | 0.0 |
| 41052 | 7.1 |
| 41053 | 3.2 |
| 41054 | 0.0 |
| 41055 | 8.2 |
| 41056 | 12.4 |
| 41057 | 9.3 |
| 41058 | 4.7 |
| 41059 | 5.4 |
| 41060 | 5.0 |
| 41061 | 8.4 |
| 41062 | 1.3 |
| 41063 | 1.1 |
| 41064 | 7.5 |
| 41065 | 0.0 |
| 41066 | 7.5 |
| 41067 | 6.9 |
| 41068 | 4.3 |Figure S1. Daily temperatures (a) and hours of sunlight (b) during the study period in the first cropping season in 2012. Gray bars indicate the period of shading treatment. The shade was removed on May 25th.

## Slide 2
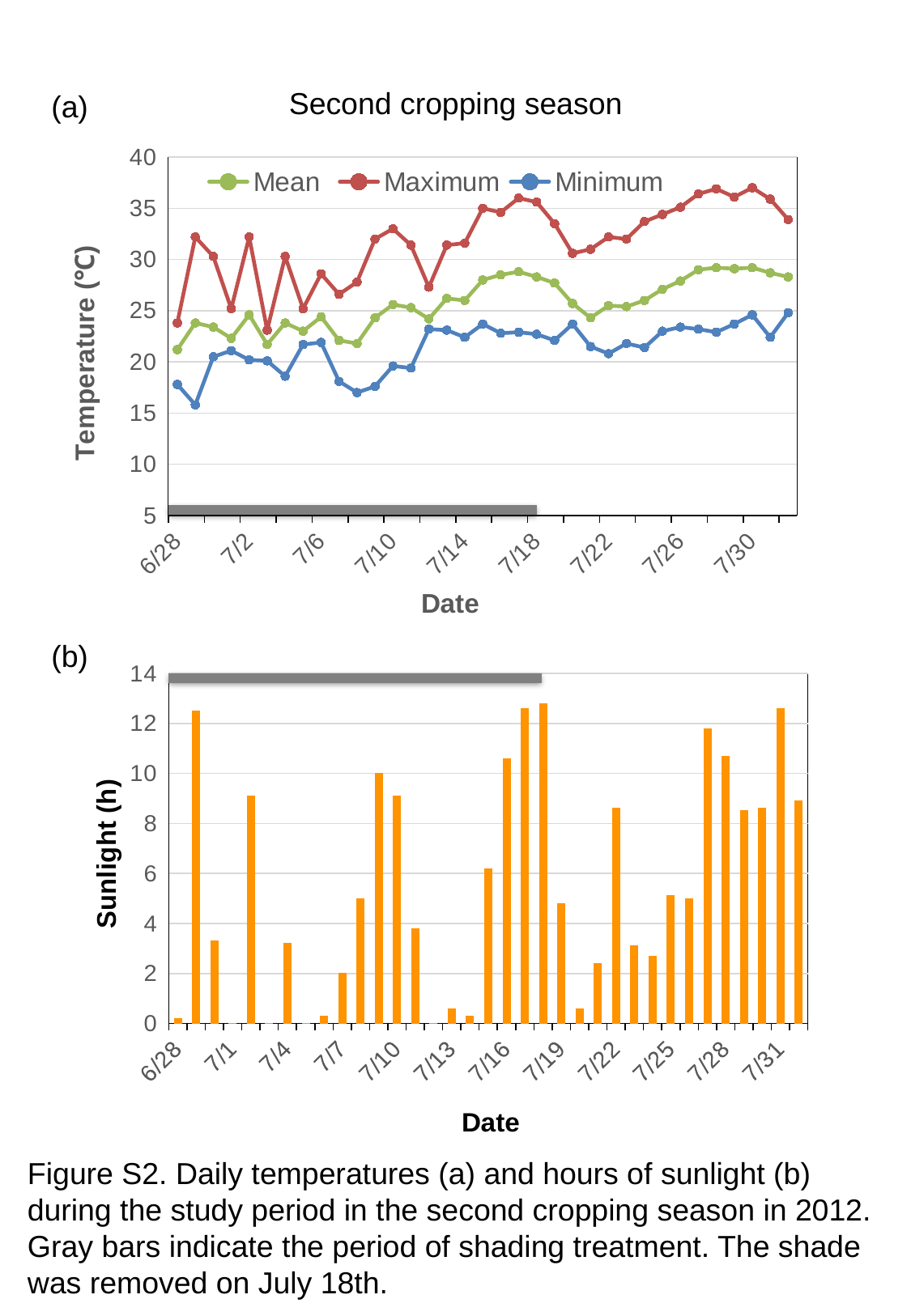

Second cropping season
(a)
### Chart
| Category | Mean | Maximum | Minimum |
|---|---|---|---|
| 41088 | 21.2 | 23.8 | 17.8 |
| 41089 | 23.8 | 32.2 | 15.8 |
| 41090 | 23.4 | 30.3 | 20.5 |
| 41091 | 22.3 | 25.2 | 21.1 |
| 41092 | 24.6 | 32.2 | 20.2 |
| 41093 | 21.7 | 23.1 | 20.1 |
| 41094 | 23.8 | 30.3 | 18.6 |
| 41095 | 23.0 | 25.2 | 21.7 |
| 41096 | 24.4 | 28.6 | 21.9 |
| 41097 | 22.1 | 26.6 | 18.1 |
| 41098 | 21.8 | 27.8 | 17.0 |
| 41099 | 24.3 | 32.0 | 17.6 |
| 41100 | 25.6 | 33.0 | 19.6 |
| 41101 | 25.3 | 31.4 | 19.4 |
| 41102 | 24.2 | 27.3 | 23.2 |
| 41103 | 26.2 | 31.4 | 23.1 |
| 41104 | 26.0 | 31.6 | 22.4 |
| 41105 | 28.0 | 35.0 | 23.7 |
| 41106 | 28.5 | 34.6 | 22.8 |
| 41107 | 28.8 | 36.0 | 22.9 |
| 41108 | 28.3 | 35.6 | 22.7 |
| 41109 | 27.7 | 33.5 | 22.1 |
| 41110 | 25.7 | 30.6 | 23.7 |
| 41111 | 24.3 | 31.0 | 21.5 |
| 41112 | 25.5 | 32.2 | 20.8 |
| 41113 | 25.4 | 32.0 | 21.8 |
| 41114 | 26.0 | 33.7 | 21.4 |
| 41115 | 27.1 | 34.4 | 23.0 |
| 41116 | 27.9 | 35.1 | 23.4 |
| 41117 | 29.0 | 36.4 | 23.2 |
| 41118 | 29.2 | 36.9 | 22.9 |
| 41119 | 29.1 | 36.1 | 23.7 |
| 41120 | 29.2 | 37.0 | 24.6 |
| 41121 | 28.7 | 35.9 | 22.4 |
| 41122 | 28.3 | 33.9 | 24.8 |(b)
### Chart
| Category | Sunlight (h) |
|---|---|
| 41088 | 0.2 |
| 41089 | 12.5 |
| 41090 | 3.3 |
| 41091 | 0.0 |
| 41092 | 9.1 |
| 41093 | 0.0 |
| 41094 | 3.2 |
| 41095 | 0.0 |
| 41096 | 0.3 |
| 41097 | 2.0 |
| 41098 | 5.0 |
| 41099 | 10.0 |
| 41100 | 9.1 |
| 41101 | 3.8 |
| 41102 | 0.0 |
| 41103 | 0.6 |
| 41104 | 0.3 |
| 41105 | 6.2 |
| 41106 | 10.6 |
| 41107 | 12.6 |
| 41108 | 12.8 |
| 41109 | 4.8 |
| 41110 | 0.6 |
| 41111 | 2.4 |
| 41112 | 8.6 |
| 41113 | 3.1 |
| 41114 | 2.7 |
| 41115 | 5.1 |
| 41116 | 5.0 |
| 41117 | 11.8 |
| 41118 | 10.7 |
| 41119 | 8.5 |
| 41120 | 8.6 |
| 41121 | 12.6 |
| 41122 | 8.9 |Figure S2. Daily temperatures (a) and hours of sunlight (b) during the study period in the second cropping season in 2012. Gray bars indicate the period of shading treatment. The shade was removed on July 18th.
